# *Pichia pastoris* composition expressed aerolysin mutant of *Aeromonas veronii* as an oral vaccine evaluated in zebrafish (*Danio rerio*)

**DOI:** 10.1007/s42995-024-00239-9

**Published:** 2024-08-20

**Authors:** Yuan-Yuan Yao, Qing-Shuang Zhang, Shu-Bin Liu, Hong-Wei Yang, Xing-Yu Chen, Ya-Lin Yang, Chen-Chen Gao, Chao Ran, Tsegay Teame, Zhen Zhang, Zhi-Gang Zhou

**Affiliations:** 1 Key Laboratory for Feed Biotechnology of the Ministry of Agriculture, Institute of Feed Research of Chinese Academy of Agricultural Sciences, Beijing, 100081 China; 2 Sino-Norway Fish Gastrointestinal Microbiota Joint Lab, Institute of Feed Research of Chinese Academy of Agricultural Sciences, Beijing, 100081 China; 3 Institute of Marine Sciences, Shantou University, Shantou, 515063 China; 4 Tigray Agricultural Research Institute (TARI) Mekelle Center, Tigray, 7101 Ethiopia

**Keywords:** *Aeromonas veronii*, Aerolysin mutant NTaer, Oral vaccine, Immune response, Gut microbiota

## Abstract

Vaccines are one of the most practical means to stop the spreading of *Aeromonas veronii* in aquaculture. In this study, virulence factor aerolysin mutant NTaer which has lost its hemolytic activity was used as a target antigen. *Pichia pastoris* constitutive secretory expression NTaer (GS115-NTaer) was used as a potential safe oral vaccine to evaluate its effectiveness on zebrafish immunity. The result shows that vaccination of GS115- NTaer for four weeks did not affect the growth performance of the host, while eliciting an effective immune protective response. Compared with the control group, the GS115-NTaer could significantly up-regulate the relative expression level of the intestinal tight junction protein 1α (*TJP1α*) gene, and significantly increased the contents of lysozyme (LYZ), complement C3 and C4 in the gut, indicating that the innate immune response of the fish was activated. The relative gene expression levels of macrophage-expressed gene 1 (*MPEG1*) and T cell receptor (*TCR-α*) in the gut, and *MPEG1*, *CD4*, *CD8*, *TCR-α*, *GATA3,* and *T-bet* in the spleen were all increased significantly, indicating that the cellular immune response of the fish was activated. Furthermore, the contents of serum IgM and intestinal mucosa IgZ antibodies were significantly increased, which showed that humoral immunity was also activated. Moreover, inoculation with GS115-NTaer significantly changed the structure of gut microbiota. In particular, the relative ratio of (Firmicutes + Fusobacteriota + Bacteroidota)/Proteobacteria was significantly higher than that of the control and GS115 groups. Lastly, the vaccinated fish were challenged with *A. veronii,* and the relative percent survival of GS115 and the GS115-NTear groups was 14.28% and 33.43%. This improvement of immunity was not only due to the specific immune response but also attributed to the improvement of innate immunity and the gut microbiota which was demonstrated by the germ-free zebrafish model. Collectively, this study provides information on the effectiveness of GS115-NTear as an oral vaccine for the green prevention and control of *A. veronii* infection in fish aquaculture.

## Introduction

High-density intensive aquaculture, which is the most widespread cultured mode, has become associated with water quality deterioration and frequent disease issues. High incidence and rapid transmission of infectious diseases have caused enormous economic losses, exceeding US$6 billion per annum. This confirms that disease has become one of the major factors in restricting the prosperity of the aquaculture industry (Stentiford et al. 2017).

*Aeromonas veronii* may cause high mortalities when water quality deteriorates and the fish become stressed. The epidemiological data showed that the most important cause of septicemia in cyprinid fish in southern China was *A. veronii* rather than *A. hydrophila* (Ran et al. 2018). *A. veronii* caused hemorrhages and skin ulcers in cultured channel catfish (*Ictalurus punctatus*) resulting in a 50–70% mortality rate (Qin et al. 2022). *A. veronii* infection was also found in crucian carp (*Carassius auratus gibelio*) (Chen et al. 2019), sea bass (*Lateolabrax maculatus*) (Wang et al. 2021) and guppy (*Poecilia reticulata*) (Lazado and Zilberg 2018). *A. veronii* could co-infect fish, livestock and humans, and carry more virulence genes. Hence, *A. veronii* infection in commercial fish farms may pose a potential threat to public health (Li et al. 2020).

Vaccines have been shown to be an effective method for the control of *A. veronii*. A live attenuated (Zhang et al. 2020) or inactivated vaccine (Song et al. 2018) for *A. veronii* increases the ability of fish to defend against bacterial invasion, but the addition of adjuvants (Song et al. 2022) or inoculation by injection is costly**.** Oral inoculation of recombinant *Lactobacillus casei* vaccine, which includes the outer membrane proteins or flagellin as antigens, led to an effective immune response in carp (Kong et al. 2019; Tian et al. 2019; Zhang et al. 2018, 2019). Antigen selection was important for the effectiveness of the vaccine, but some antigens, such as secreted protein (AcrV) (Kong et al. 2020) and Aha1 (Chen et al. 2022a, b), from *A. hydrophila* were referred to as the antigen in *A. veronii*. As the key virulence factor in *A. veronii*, aerolysin could disrupt the intestinal barrier of the host, and contributed to the invasion of aeromonads in single or mixed infection, whereas it does not protect the pathogen from the host immune system (Ran et al. 2018). Therefore, aerolysin is regarded as a good candidate antigen. Moreover, yeasts are excellent hosts for the expression of functional recombinant proteins in medical or industrial fields (Baghban et al. 2019).

In this study, a mutant of aerolysin NTaer, which lost its hemolytic toxicity, was used as an antigen. The constitutive secretory expression of NTaer in *Pichia pastoris* was constructed as an oral vaccine. The safety and immunoprotective effect induced by this oral vaccine was evaluated, and the results of this study may provide information on the effectiveness of GS115-NTaer to prevent and control infections by *A. veronii* in aquaculture.

## Materials and methods

### Bacterial strains and growth conditions

Commercialized *P. pastoris* GS115 (Coolaber) were used for constructing recombinant strains, which could constitutively secrete aerolysin mutants of *A. veronii* as oral vaccines. The screened positive clones of nutrient-deficient *P. pastoris,* which was transformed with expressed plasmid, using minimal dextrose (MD) agar medium. Proliferations of positive *P. pastoris* clones were carried out routinely using yeast extract peptone dextrose (YPD) broth medium at 30 °C for 24 h. Construction of the recombinant plasmid was carried out in *Escherichia coli* strain DH5α, which was cultured in Luria–Bertani (LB). The pathogenic *A. veronii* Hm091, which was isolated from silver carp, was grown in LB broth medium for 18 h at 37 °C and used to to challenge zebrafish (Ran et al. 2018).

### Construction and confirmation of recombinant the *P. pastoris* strain

Commercial plasmid pPIC9 (Huayueyang) was used as the initial vector, and glyceraldehyde 3-phosphate dehydrogenase (GAPDH) protein-coding gene promoter (P_GAP_) was used to replace the original alcohol oxidase (AOX1) protein-coding gene promoter. Secretory signal peptide sp23 was used to replace the original a-factor secretion signal peptide. The aerolysin (NCBI reference sequence: WP_236322878.1) deletion mutant NTaer (contains amino acid sequence from 107 to 488) was used as an antigen, and eight histidine tags were added to the C-terminal of the NTaer protein to facilitate the detection of the target protein expression; the final plasmid was named as pGAP-SP23-NTAer. Plasmid pGAP-SP23-NTAer was linearized using *Sal*I (NEB) digestion, and the fragments were recovered by agarose gel electrophoresis, and then were chemically transformed into commercialized *P. pastoris* GS115 competent cells (Coolaber). For specific methods, refer to GS115 competent cell instruction manual. The transformed GS115 was spread onto MD plates with incubation at 30 °C for 48–72 h. Then a single colony was picked and cultured in YPD for 24 h, and the yeast genome was extracted for PCR with primers GAP-F (5′-ACGCATGTCTTGAGATTATTGGA-3′) and 3AOX-R (5′-GGCAAATGGCATTCTGACATCCT-3′). The instruction of 2× *Taq* PCR StarMix with Loading Dye (GenStar) was used in a 20 μL PCR reaction system. The positive clone GS115-NTaer was cultured in fresh YPD broth for 48 h, and a 1 mL volume of supernatant was collected. The protein was precipitated by acetone at −80 °C for 30 min, and then the precipitant was re-suspended with 100 μL of phosphate-buffered saline (PBS). The Western blot (WB) was used to verify the successful expression of NTaer. The specific method of WB was as follows: concentrated protein samples were separated by 12% SDS-PAGE and transferred to polyvinylidene difluoride membranes (Bio-Rad). The membranes were blocked for 1 h at room temperature in TBST buffer (25 mmol/L Tris–HCl, 150 mmol/L NaCl and 0.1% Tween 20 [pH 7.5]) containing 5% nonfat dry milk (BD) and probed with a mixture of anti-his monoclonal antibody (TianGen, dilute at 1:1000) and Goat anti-mouse IgG (Abbkine, dilute at 1:5000) overnight at 4 °C. Washing was for three times with TBST, and then the membranes were stained with Immobilon TM Western Chemiluminescent HRP Substrate (Millipore) and detected using an ChemiDoc™ MP Imaging system (Bio-Rad).

### Hemolytic analysis

The recombinant aerolysin of *A. veronii* named Hmaer (Ran et al. 2018) and its mutant NTaer with a polyhistidine tag were purified by nickel resin (Beyotime). Three microliters (μL) of 0.1 mg/mL recombinant aerolysin Hmaer, mutant NTaer and PBS as control were dropped vertically onto a blood agar plate and incubated at 37 °C for 48 h.Then, the safety of the mutant NTaer was further verified by the hemolysis rate. Ten milliliter (mL) of fresh sheep blood was centrifuged at 1000 r/min for 5 min, the supernatant was removed, and the precipitated red blood cells were washed three times with normal saline. Then, red blood cells were prepared as a 2% suspension with normal saline. Next, 0.9 mL of Hmaer or NTaer protein solution with a concentration of 1 mg/mL, 2.5 mg/mL, 5 mg/mL, 10 mg/mL and 20 mg/mL were added to 0.1 mL of the red blood cell suspension. Normal saline was added as the negative control, and sterile water was added as the positive control. After gently mixing, and incubating at 37 °C for 1 h, centrifugation was at 1000 rpm/min for 5 min. Supernatant in 300 μL volumes was pipetted into the wells of a 96-well microtiter plate, and the absorbance ws measured at 540 nm using a microplate reader (BioTek). Three replicates were used for each concentration of protein; the % hemolysis rate was the experimental group value − negative control value)/(positive control value − negative control value) × 100%.

### Vaccination

Vaccination was carried out in zebrafish with a similar initial body weight (~ 0.032 g). The fish were randomly assigned to three groups, each with ~ 120 fish in six replicates. Three groups were fed respectively with basal diet as control, diets supplemented with *P. pastoris* GS115 strain (final concentration of yeast was ~ 5 × 10^6^ cfu/g) and recombinant *P. pastoris* GS115-NTaer strain (final concentration of yeast was ~ 5 × 10^6^ cfu/g). The control diet was prepared by proportionally weighing and mixing with different ingredients (see Table 1); soybean oil was added finally. GS115 and GS115-NTaer strains were cultured in YPD broth and incubated 30 °C for 48 h with shaking (200 r/min), and the number of yeast cells was determined by plate counts. Then, suitable volumes of fermentation products were added with water during feed preparation to the specified yeast concentration to obtain the medicated feed; the error of nutrient contents in the medicated feed was less than 1% compared with the control feed. The vaccine was administered orally. The feeding experiments were carried out in a recirculating water system (at 60 L/h); the fish were fed twice daily at 6% of body weight; then increased by 1% after a week. The water was turned off until the feed was eaten; the vaccination lasted for four weeks. The experimental water parameters were a water temperature at 26 °C, dissolved oxygen of > 6.0 mg/L, pH at 7.0–7.2, nitrite content of < 0.005 mg NO_2_-N/L, and ammonia nitrogen of < 0. 2 mg NH_3_-N/L.

**Table 1:** The ingredients and nutrient composition of the control diet

Ingredients (g/kg)	Control diet
Casein	400
Gelatin	100
Dextrin	280
Bean oil	60
Lysine	3.3
VC lecithin	1
Vitamin premix	4
Mineral premix	4
Calcium dihydrogen phosphate	20
Choline chloride	2
Sodium alginate	20
Microcrystalline cellulose	40
Zeolite powder	65.7
Total	1000
nutrient composition	
Crude protein	421.9
Crude lipid	60.1

### Sampling and challenge test

At the end of the four-week immunization period, the number of fish, feed used, and final weight of fish were measured for each tank. Then, weight gain, feed conversion ratio (FCR), and survival rate were calculated. Zebrafish were euthanized with 0.2% Tricaine, and the hindgut was collected to detect the expression of immune-related genes by qRT-PCR, and was also used to detect the lysozyme, complement C3, C4, and IgZ after homogenization. The spleen was collected and used to detect the expression of immune-related genes by qRT-PCR. The caudal fin of zebrafish was punctured to collect blood, and serum was prepared by centrifugation at 4,000 *g* for 10 min (4 °C). The serum was used to detect IgM. Gut content samples collected 6 h after the last feeding were used to analyze gut microbiota composition and diversity. Samples from 2–3 fish were randomly taken and mixed together. There were six replicates in each group, and the samples were frozen at -80 °C before analysis.

Post-vaccination, the immunized fish were challenged with *A. veronii*. Thirty fish from each group were randomly assigned to three tanks one day before the challenge to acclimate. The monoclones of *A. veronii* Hm091 were selected into 60 mL LB broth and incubated at 37 °C for 18 h with shaking at 200 r/min. Then, the number of bacterial cells was determined by plate counting. The final concentration of *A. veronii* in the immersion challenge was ~ 5 × 10^8^ cfu/mL, which was determined by means of a median lethal dose (LD50) and the experience of multiple challenges. Dead fish were removed promptly, and the survival rate was calculated with "1" representing "death" and "0" representing "survival". Observation continued until the fish stabilized and no more deaths occurred. The relative percent survival (RPS) was calculated according to Amend (1981).

### Quantitative real-time RT-PCR

Total RNA was extracted by TRIZOL Reagent (Invitrogen), and then referred to fastKing gDNA dispelling RT superMix kit (TianGen) instructions for cDNA synthesis and contaminated genomic DNA elimination. qRT-PCR was performed using SYBR Green Supermix (TianGen) on a Light Cycler 480 (Roche 480) to test the expression of reference gene β-actin and target gene. qRT-PCR conditions were as follows: 95 °C for 5 min and then 45 cycles of 95 °C for 20 s, 60 °C for 20 s, and 72 °C for 20 s. The final result is shown as the relative expression of mRNA compared to the control group using the 2^−ΔΔCt^ method (Livak and Schmittgen 2001). The primers for each target gene are given in Table 2.

**Table 2:** List of qRT-PCR primers used in this study

Gene	Forward primer (5′-3′)	Reverse primer (5′-3′)	Size (bp)	Accession no.
*β-actin*	ATGGATGAGGAAATCGCTG	ATGCCAACCATCACTCCCTG	140	AF057040.1
*Occludin-b*	TGGAGATGAGCTTGACACAGATG	CCTTCCTCTAGCCTGTCGAG	177	BC164682.1
*Claudin7*	GGAGCCCTCTGTAGCATTGTT	ATTAGGAGATGACTGACCCTTTGA	198	BC066408.1
*Claudin11a*	ACCAGAAAGTGCCAAGAACA	AAAGCCAAAGGACATCAGACC	136	NM_001002624
*TJP1a*	CAAAGACCAACAGCACTGCC	GTGGTTTAGCGGTGATGGGA	177	XM_021477863
*C4*	AGGGAACAGGACGGGGAAC	GTCGGTTTTGTACTCCAGCTCT	125	XM_005157372.4
*C3a*	ATGAGCTCCTGCAGAGGTGT	AGTGGTTGTTGGAGGTCTGG	179	XM_005174032
*LYZ*	GATTTGAGGGATTCTCCATTGG	CCGTAGTCCTTCCCCGTATCA	103	NM_139180
*MHCII*	ACAGGGACTGATATTTGCA	CAGCCTGCCTCACTGACTTG	167	NM_131476
*CD4*	GTGTTTGGACATGCCAGTTG	AAGCACAGGGAATGCTGACT	144	HE983359.2
*CD8α*	AGGTTGTGGACTTTTCCTCGT	GGAGCTAGAAGTGGCTGGTG	149	BC162235.1
*T-bet*	GCAGCTCCAACAACGTAGCA	ATCCTCCTTCACCTCCACGATG	89	NM_001170599.2
*GATA3*	GGTGAGATGTAGGGAGAGGAAACC	TGCCCAAGACCTATAACACATCCA	120	XM_005164809
*TCRα*	CTTAAAACGTCGGCTGTCCG	TGAACAAACGCCTGTCTCCT	93	AY476727.1
*MPEG1*	ATGTGGATTCCCCAAACTTCAACT	TGGTAAATGCCACCAAAGCTAAGA	94	CP137045.2

### The detection of immunological index

Lysozyme (LYZ), C3, C4, IgM, and IgZ of zebrafish were detected by the ELISA (Jiangsu Meimian Industrial Co., Ltd. China). The test was carried out according to the manufacturer’s instructions. The basic principle for lysozyme, C3, and C4 was the double-antibody sandwich method; IgM and IgZ were examined by the double-antigen sandwich method.

### Analysis of intestinal microbiota composition

The sequencing analysis of the intestinal microbiota was carried out by BioMarker Technologies. The total DNA of digested samples was extracted using a TIANamp bacterial DNA kit (TianGen). Then, the conserved V3–V4 region of 16S rRNA was amplified by F: 5′-ACTCCTACGGGAGGCAGCA-3′; R: 5′-GGACTACHVGGGTWTCTAAT-3′. The qualified products were used to build the sequencing libraries, which were sequenced by Illumina NovaSeq 6000. The filtered original reads were used to identify and remove primer sequences using Cutadapt 1.9.1 software to obtain clean reads, and the final effective data (non-chimeric read) were further obtained through de-noising using dada2 method in QIIME2 2020.6. The qualified data were used for cluster analysis and diversity analysis. Principal coordinates analysis (PCoA) was applied to assess the beta diversity of gut bacterial communities based on QIIME.

### Production and treatment of germ-free zebrafish

Germ-free (GF) zebrafish were produced according to reported protocols (He et al. 2017). GF zebrafish were treated on 5 dpf, and two experiments were carried out. Experiment 1 was about GS115 or GS115-NTaer strain (final concentration 10^6^ cfu/mL) directly immersed GF zebrafish; the untreated group served as control. Experiment 2 was gut microbes from three groups of feeding experiments (control, GS115 and GS115-NTaer) to immerse GF zebrafish. Simply, intestinal contents of five fish were sampled 4 h after the last feeding at the end of the feeding experiment. After being fully mixed, the impurities were removed by low-speed centrifugation and bacteria with the final concentration ~ 10^6^ cfu/mL were added to the GF zebrafish culture flask. Each experimental group contained ten replicates; each replicate comprised 30 zebrafish larvae in a sterile culture flask containing 30 mL gnotiobiotic zebrafish media. After three days of treatment of GF zebrafish larvae, each group of ninety fish was divided into three replicates to challenge with *A. veronii*. From each treatment group, thirty larvae were collected into a single sample, and six replicates were used for RNA extraction and gene expression detection.

### Statistical analysis

Data were analyzed by one-way analysis of variance (ANOVA) with Duncan’s post hoc test in IBM SPSS Statistics, and the survival data after *A. veronii* challenge was analyzed by the Log-rank (Mantel-Cox) test. A statistically significant difference was when *P* < 0.05, and *P* between 0.05 and 0.1 was considered a trend. Figures were prepared in GraphPad Prism Version 8 software.

## Results

### Construction of the recombinant *P. pastoris* GS115-NTaer strain

**Fig. 1:**
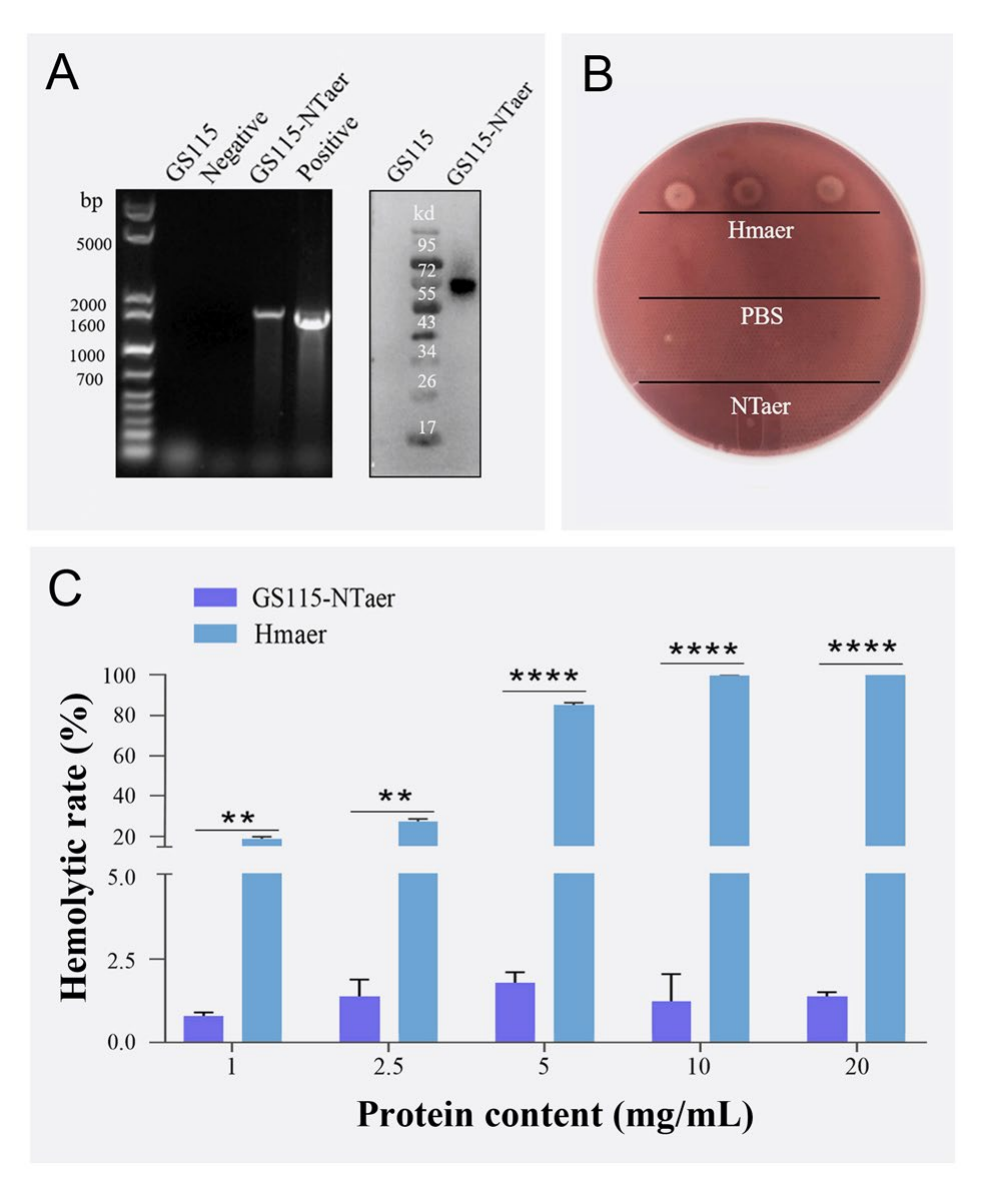
Validation of the recombinant *P. pastoris* GS115-NTaer strain and safety of the antigen. **A** PCR analysis of *P. pastoris* genome and WB analysis of proteins secreted into medium. **B** Hemolysis test on blood plate. **C** Hemolysis rate of different concentrations of aerolysin Hmaer and its mutant NTaer. In two-group comparisons, significant differences were marked with ** (*P* < 0.01) or **** (*P* < 0.0001)

First, the recombinant strains were verified by genomic PCR. As shown in Fig. 1A, GS115 and the negative control had no target bands, whereas a band with the same size (~ 1500 bp) as the positive plasmid was amplified in the genome of the selected recombinant clone. It indicated that the antigen gene was successfully transferred into GS115. Second, the expression of antigen was verified by western blotting, and a single target band was detected in the supernatant of the yeast culture of the GS115-NTaer recombinant strain but not in the GS115 strain, indicating that the antigen protein was successfully secreted. Third, the safety of antigen was evaluated. In vitro, the aerolysin Hmaer of *A. veronii* Hm091 revealed hemolytic rings on the blood plate, but its mutant NTaer and PBS control were not hemolytic (Fig. 1B). Further experimental results of hemolysis rate showed that the mean hemolysis rate of Hmaer was significantly higher than that of NTaer at different protein concentrations (Fig. 1C).

### Vaccination did not significantly affect the growth of the host

**Fig. 2:**
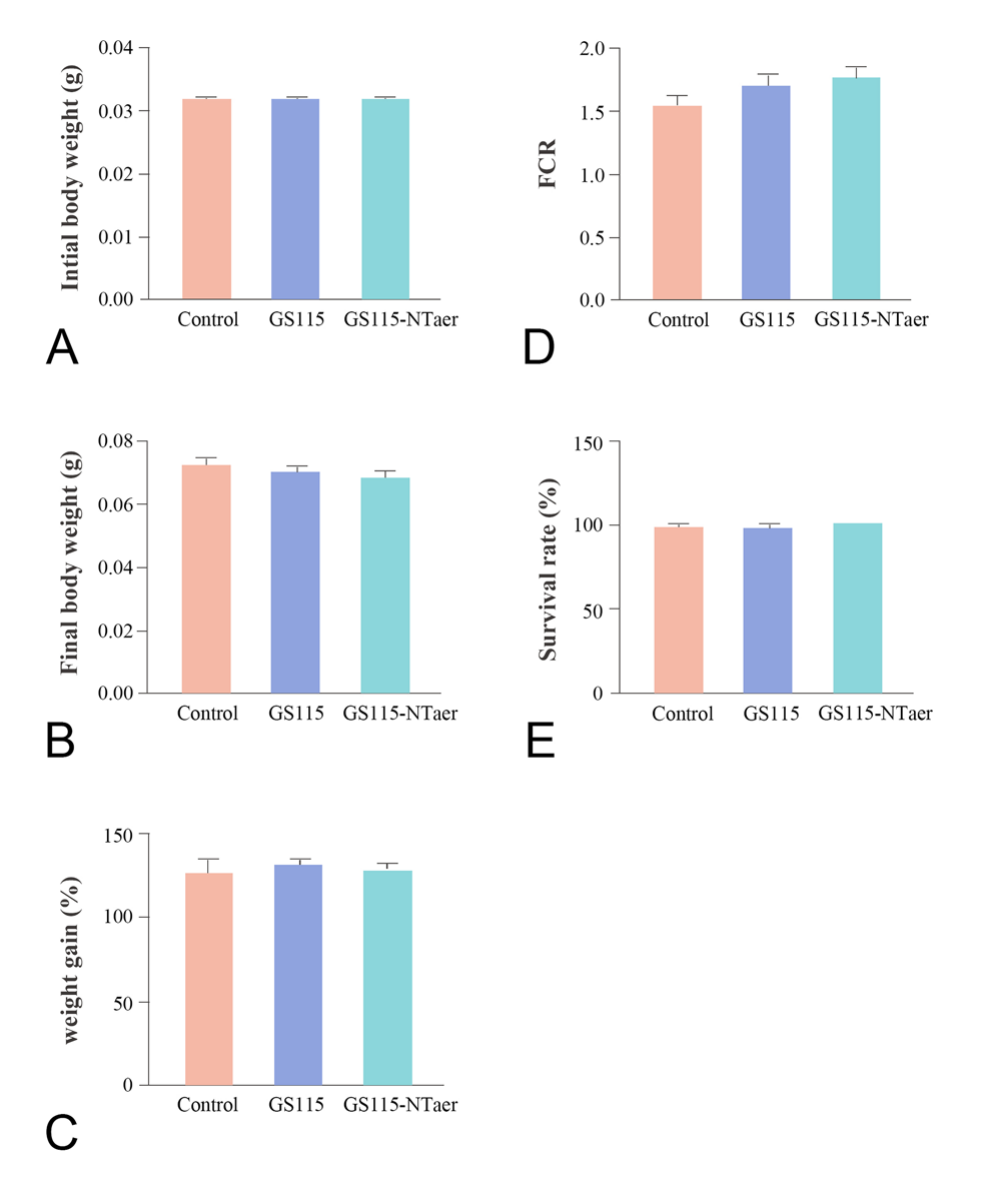
Growth performance of zebrafish fed with control diet, GS115 or GS115-NTaer vaccine. **A** Initial body weight. **B** Final body weight. **C** Weight gain (%). **D** Feed conversion rate (FCR). **E** Survival rate (%). Data represent the means (± SD) of six replicates of each group, significant differences would be marked with different letter when *P* < 0.05

At the start of the feeding experiment, the initial body weights of fish in three groups were the same, ~ 0.032 g per fish (Fig. 2A). After four weeks of oral immunization, there was no significant difference in final body weight among control, GS115 and GS115-NTaer groups (Fig. 2B), nor were there any significant differences in calculated weight gain rate (Fig. 2C) and feed conversion ratio (FCR) (Fig. 2D). Even the survival rate datum indicated no significant difference among the groups, at 97.5% for the control and GS115 groups, and 100% from the GS115-NTaer group (Fig. 2E).

### Effect of vaccine on gut epidermal physical barrier and immunological responses

**Fig. 3:**
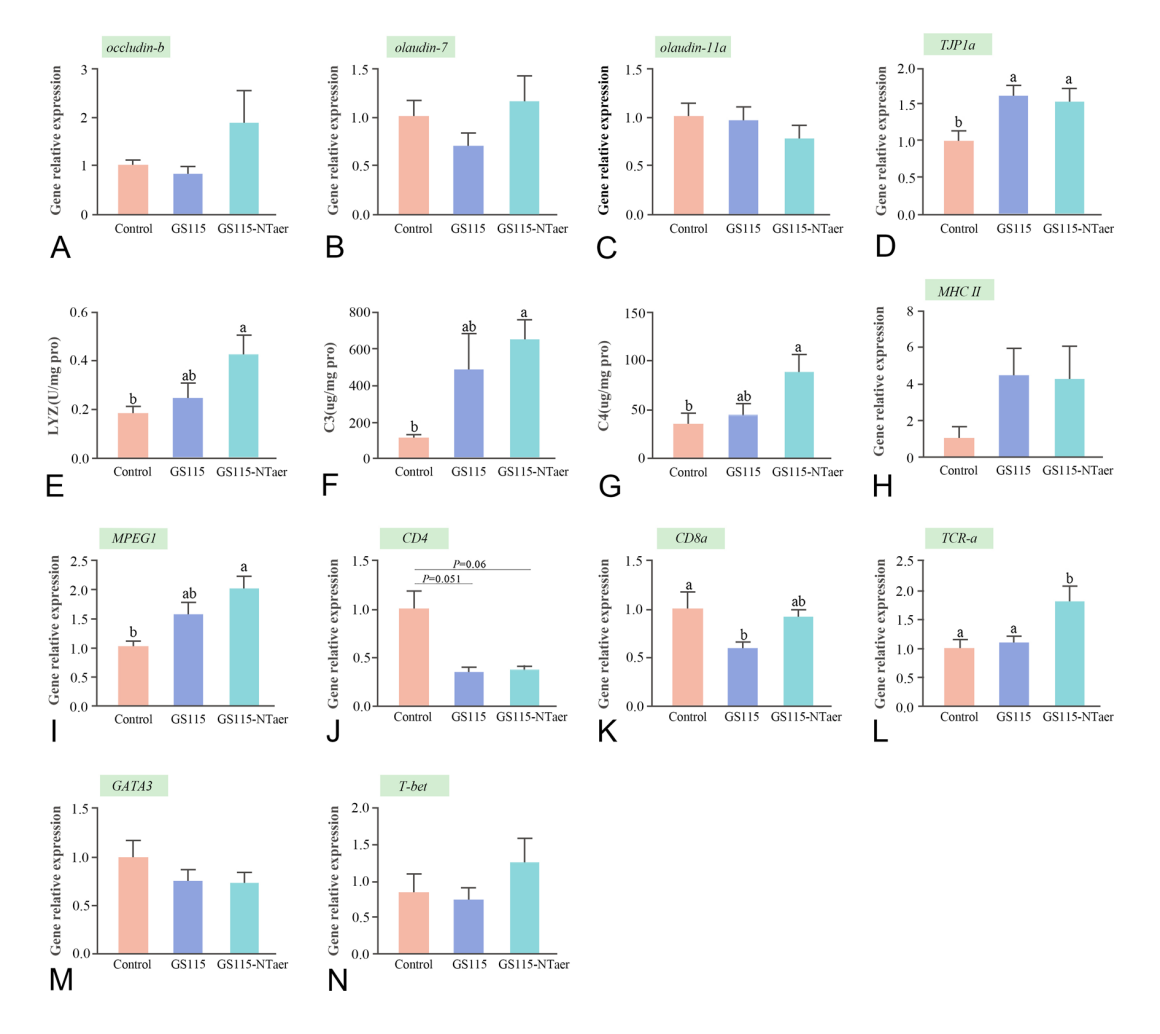
Effect of vaccine on gut epidermal physical barrier and immunological response. **A–D** Relative expression of genes associated with tight junctions. **E–G** Content of lysozyme (LYZ), complement C3 and C4 in gut. **H–N** Profile of immune-related gene expression post vaccination. Data represent the means (± SD) of six replicates of each group, groups with different letters indicate significant differences (*P* < 0.05),* P* between 0.05 and 0.1 was considered trend

In the gut, the transcription level of *occludin-b* (Fig. 3A), *claudin-7* (Fig. 3B), and *claudin-11α* (Fig. 3C) showed no obvious differences among the three groups. The expression of tight junction protein *TJP1*α was up-regulated in GS115 and GS115-NTaer groups (*P* < 0.05) when using the control group as a reference for comparison (Fig. 3D).

The contents of lysozyme (LYZ) and complement C3 and C4 in the GS115-NTaer group were increased markedly (*P* < 0.05) as compared with the controls, but there was no significant difference when compared with the GS115 group. Also, the contents of LYZ, C3 and C4 in the GS115 group were a little higher than that in the control group, without significant differences (Fig. 3E–G).

In the gut, as shown in Fig. 3H, M, and N, the mRNA levels of major histocompatibility complex II (*MHC II*), transcription factors *GATA3* and *T-bet* had no significant variation in the three groups. The relative expression of macrophage-expressed gene 1 (*MPEG1*) in the GS115-NTaer group was markedly up-regulated (*P* < 0.05; Fig. 3I) when using the control group as reference for comparison. However, in the GS115 group, there was not any significant difference as compared with the other two groups. Although there were no significant changes in the relative expression level of cluster of differentiation 4 (*CD4*) gene among the three groups, there was a downward trend in both GS115 and GS115-NTaer groups compared with the controls (Fig. 3J). For the expression of *CD8α*, its relative expression level in the GS115-NTaer group showed no significant changes compared with GS115 and the controls, but the expression level in the GS115 group was significantly reduced compared with the controls (*P* < 0.05; Fig. 3K). The expression level of the T cell receptor (*TCR-α*) gene was significantly increased in GS115-NTaer group compared with the control and GS115 groups (*P* < 0.05; Fig. 3L).

### Profile of gene expression of the spleen

**Fig. 4:**
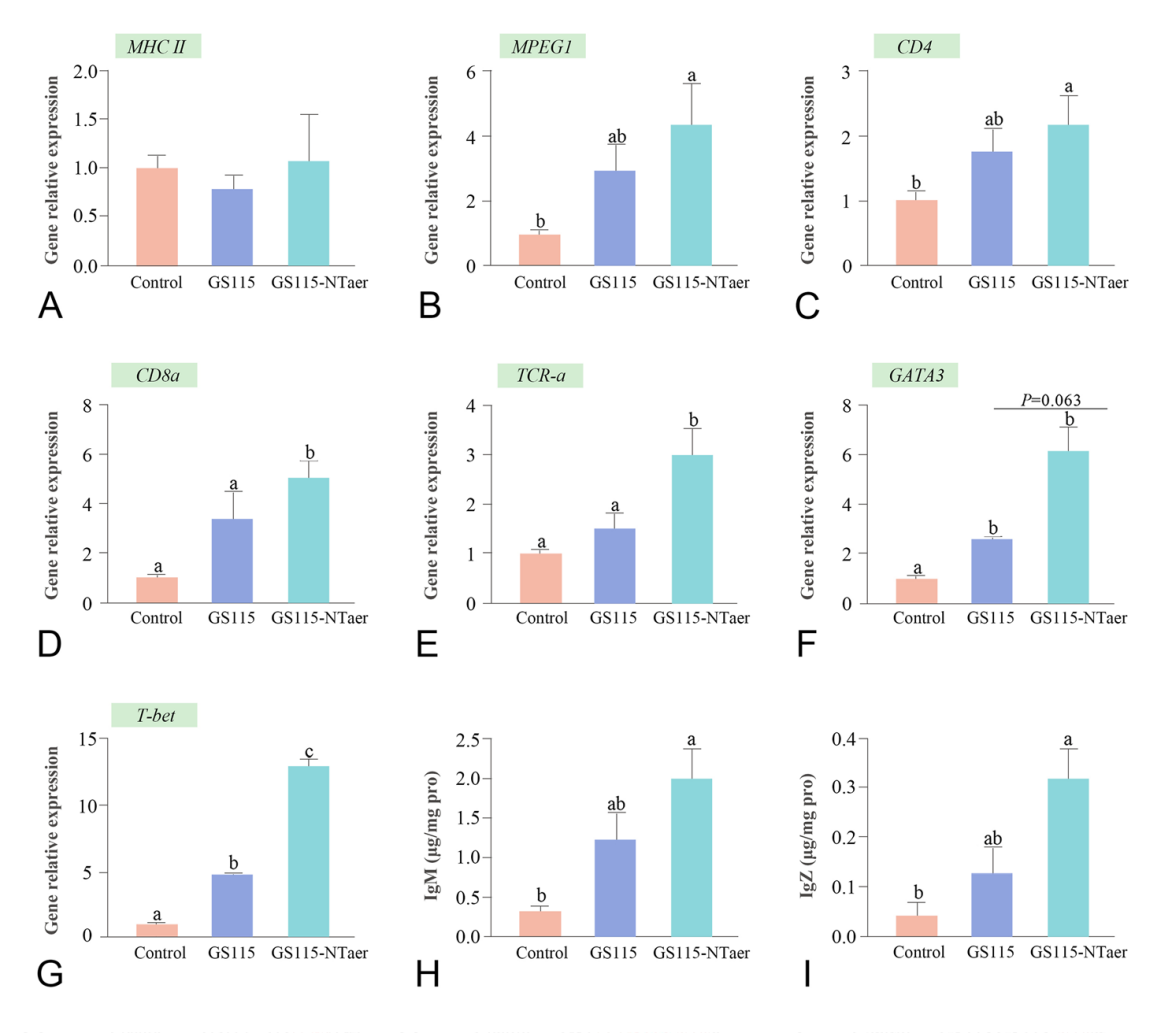
Profile of immune-related gene expression in spleen post vaccination and antibody response of immunized zebrafish. **A**–**G** Relative expression of immune-related gene expression in spleen. **H** Serum IgM content. **I** Intestinal IgZ content. Data represent the means (± SD) of six replicates of each group, groups with different letters indicate significant differences (*P* < 0.05). *P* between 0.05 and 0.1 was considered trend

In the spleen, as shown in Fig. 4A, there was no remarkable difference in the relative expression of *MHC II* among the three groups. The gene expressions of *MPEG1* and *CD4* were increased significantly in the GS115-NTaer group (*P* < 0.05) compared with the control groups (*P* < 0.05). Moreover, there was no significant change in the expression of these two genes in the GS115 group compared with the control and GS115-NTaer groups (Fig. 4B, C). The mRNA level of *CD8α* and *TCR-α* were significantly up-regulated in the GS115-NTaer group compared with both GS115 and control groups (*P* < 0.05; Fig. 4D, E). The relative expression level of *GATA3* in group GS115-NTaer had an up-regulated trend compared with GS115, and significantly up-regulated compared with the controls (*P* < 0.05). The change of *GATA3* expression level in group GS115 was also significantly up-regulated compared with the controls (*P* < 0.05; Fig. 4F). The relative expression level of *T-bet* was significantly different among the three groups (*P* < 0.05; Fig. 4G).

### Antibody response of immunized zebrafish

As shown in Fig. 4H and I, using the control group as reference, serum immunoglobulin M (IgM) and intestinal mucosal antibody IgZ levels were significantly increased in the GS115-NTaer group (*P* < 0.05). Moreover, the content fluctuation of IgM and IgZ was not apparent in GS115 compared with controls and GS115-NTaer groups.

### Vaccination improved intestinal microbiota

**Fig. 5:**
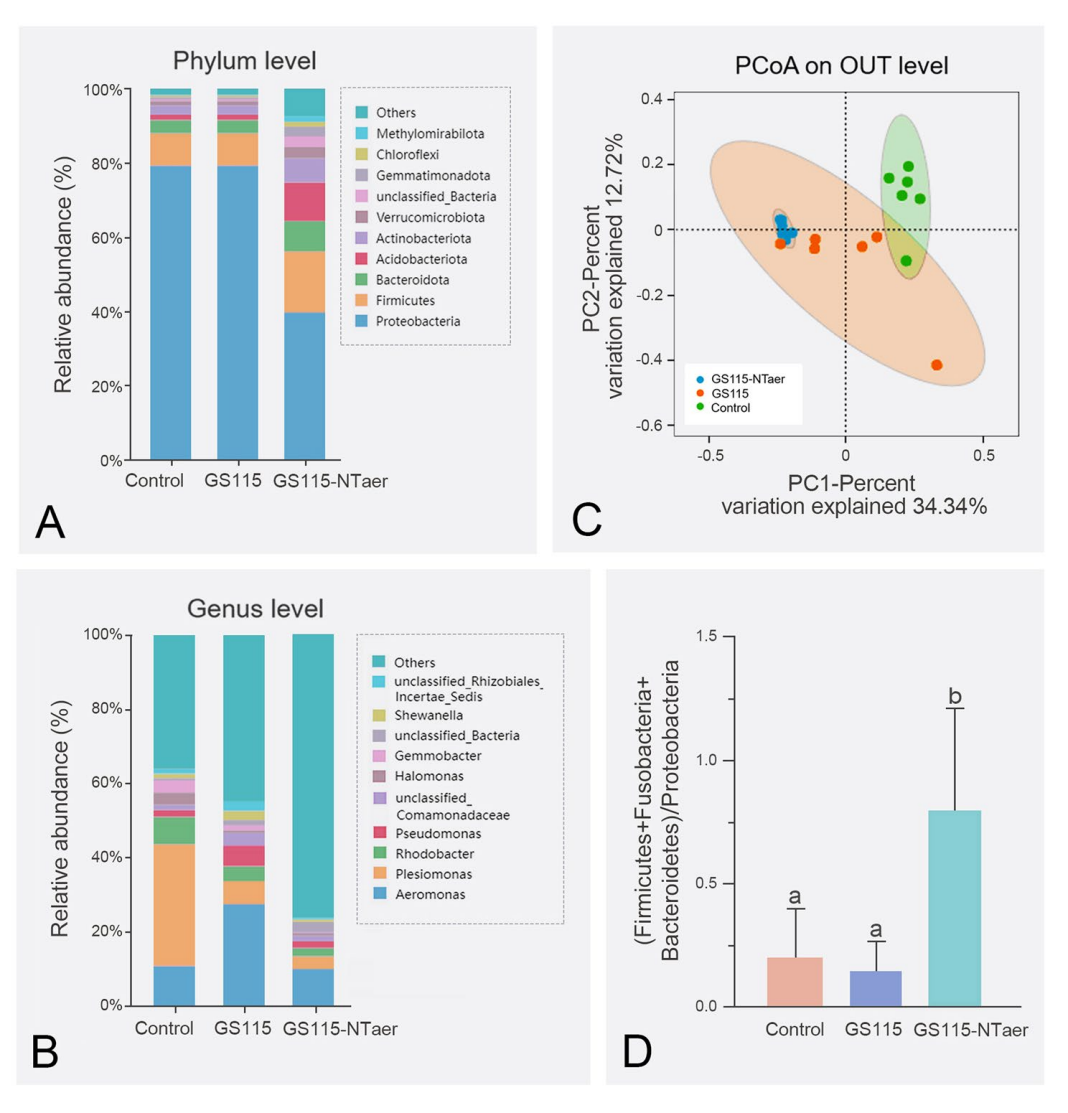
Vaccination improved intestinal microbiota. The relative bacterial abundance at the phylum (**A**) and genus level (**B**). **C** PCoA of the intestinal microbiota on OTU level. **D** The ratio of (Firmicutes + Fusobacteria + Bacteroidetes)/Proteobacteria. Groups with different letters indicate significant differences (*P* < 0.05)

The result of intestinal microbiota composition analysis showed that at the phylum level (Fig. 5A), the microbiota structure of GS115 group was similar to that of the controls without significant changes. However, in the fish vaccinated with GS115-NTaer, there was a significant reduction in the abundance of Proteobacteria, and significant increase in Firmicutes, Bacteroidota, Acidobacteriota, Actinobacteriota, and Verrucomicrobiota (*P* < 0.05). At the genus level, as illustrated in Fig. 5B, although there were changes in the microbiota community structure between GS115 and the control groups, the disparity was insignificant, except for the abundance of *Halomonas* in the GS115 group, which showed a downward trend. The difference between the GS115-NTaer and the control groups was large. The abundance of *Plesiomonas* and *Halomonas* in the GS115-NTaer group tended to decrease compared with the control group, whereas the abundance of other groups increased significantly in GS115-NTaer compared with the controls (*P* < 0.05). The abundance of *Gemmobacter* differed significantly between the GS115 and GS115-NTaer groups (*P* < 0.05), and *Shewanella* showed a decreasing trend in the GS115-NTaer group compared to GS115. PCoA analysis of the gut microbiota showed that at the OTU level, the structure of the microbiota in the GS115 and GS115-NTaer groups were different from the control group (Fig. 5C). (Firmicutes + Fusobacteria + Bacteroidetes)/Proteobacteria was used as an indicator of homeostasis of the intestinal microbial barrier, and this index was significantly higher in the GS115-NTaer group compared with the control and GS115 groups (*P* < 0.05; Fig. 5D).

### Protective efficacy of the oral vaccine

**Fig. 6:**
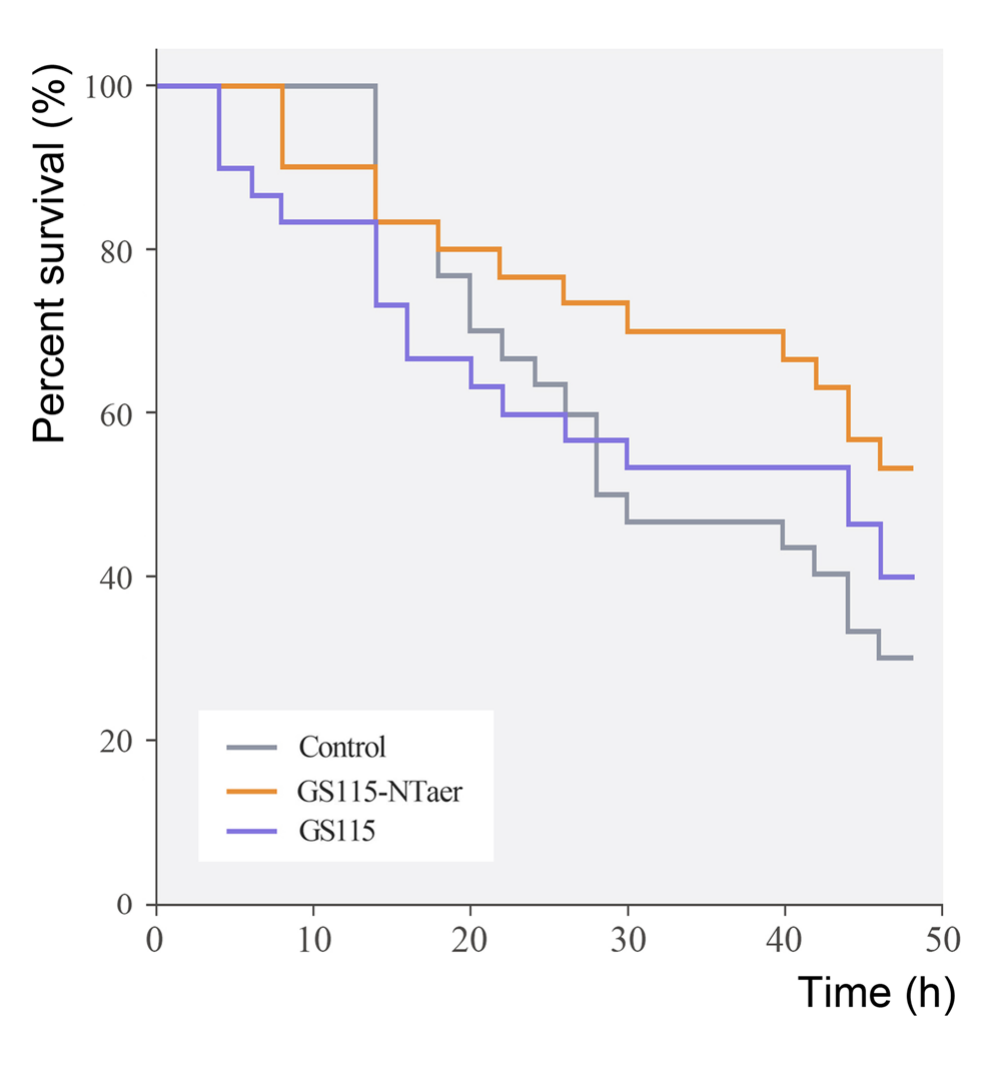
The survival curves of zebrafish after challenged by *A. veronii*

Within 48 h after *A. veronii* Hm091 challenge, the status of zebrafish in each group was stable, and deaths were not recorded. The survival graph was shown in Fig. 6. Thus, the survival of the GS115-NTaer immunized group was better than that of controls throughout the challenge. Moreover, the final survival rate for the controls, GS115 and GS115-NTaer was 30%, 40%, and 53.4%, respectively. Hence, the corresponding RPS of GS115 and GS115-NTaer was 14.28% and 33.43%.

### Effect of the direct and gut microbiota-mediated roles of vaccine in the GF zebrafish model

**Fig. 7:**
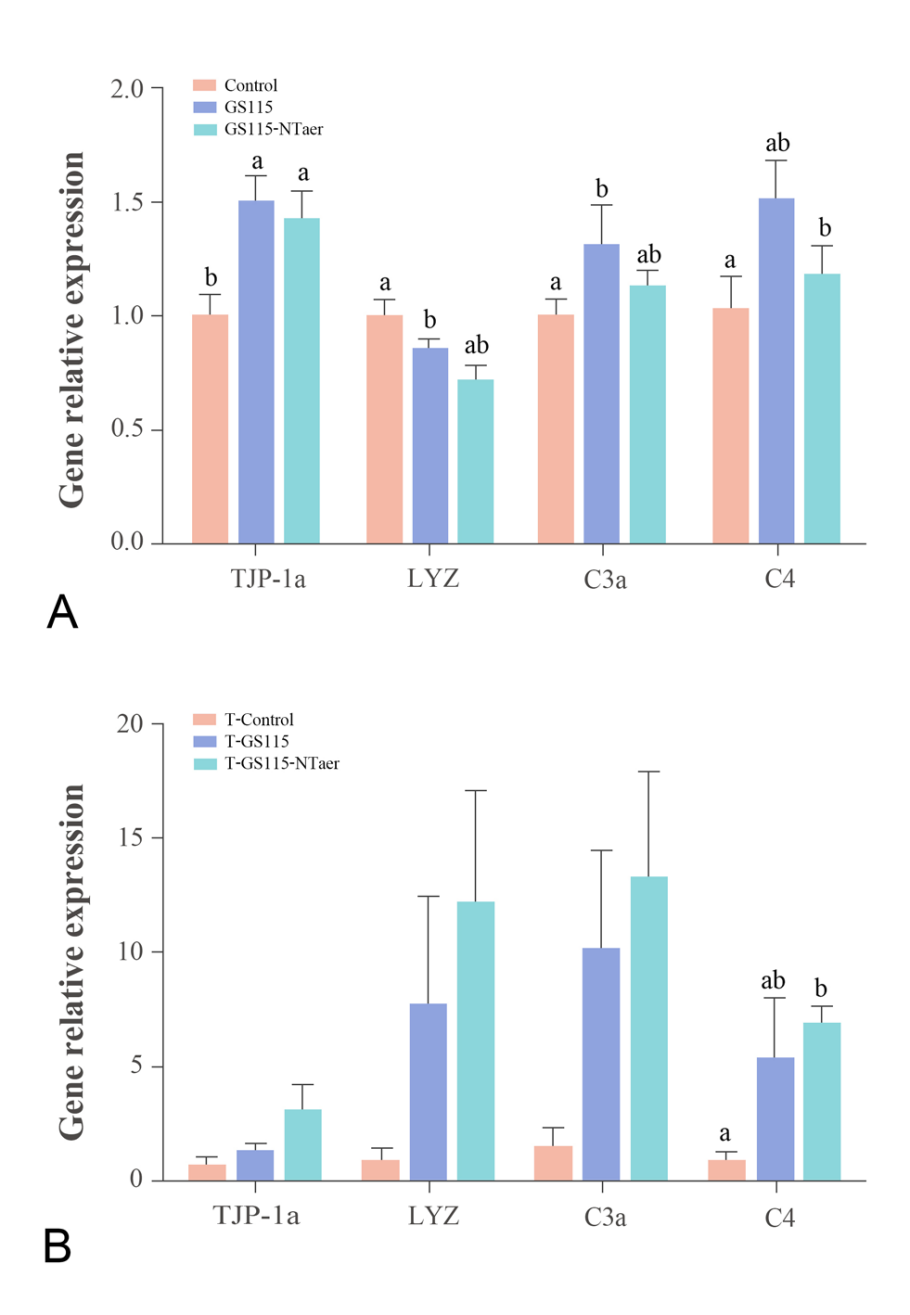
Gene expression of germ-free zebrafish. Gene expression induced by directly immersed germ-free zebrafish with GS115 or GS115-NTaer strain (**A**) and by intestinal microbiota caused by control, GS115, or GS115-NTaer vaccine diet (**B**). Groups with different letters indicate significant differences (*P* < 0.05)

**Fig. 8:**
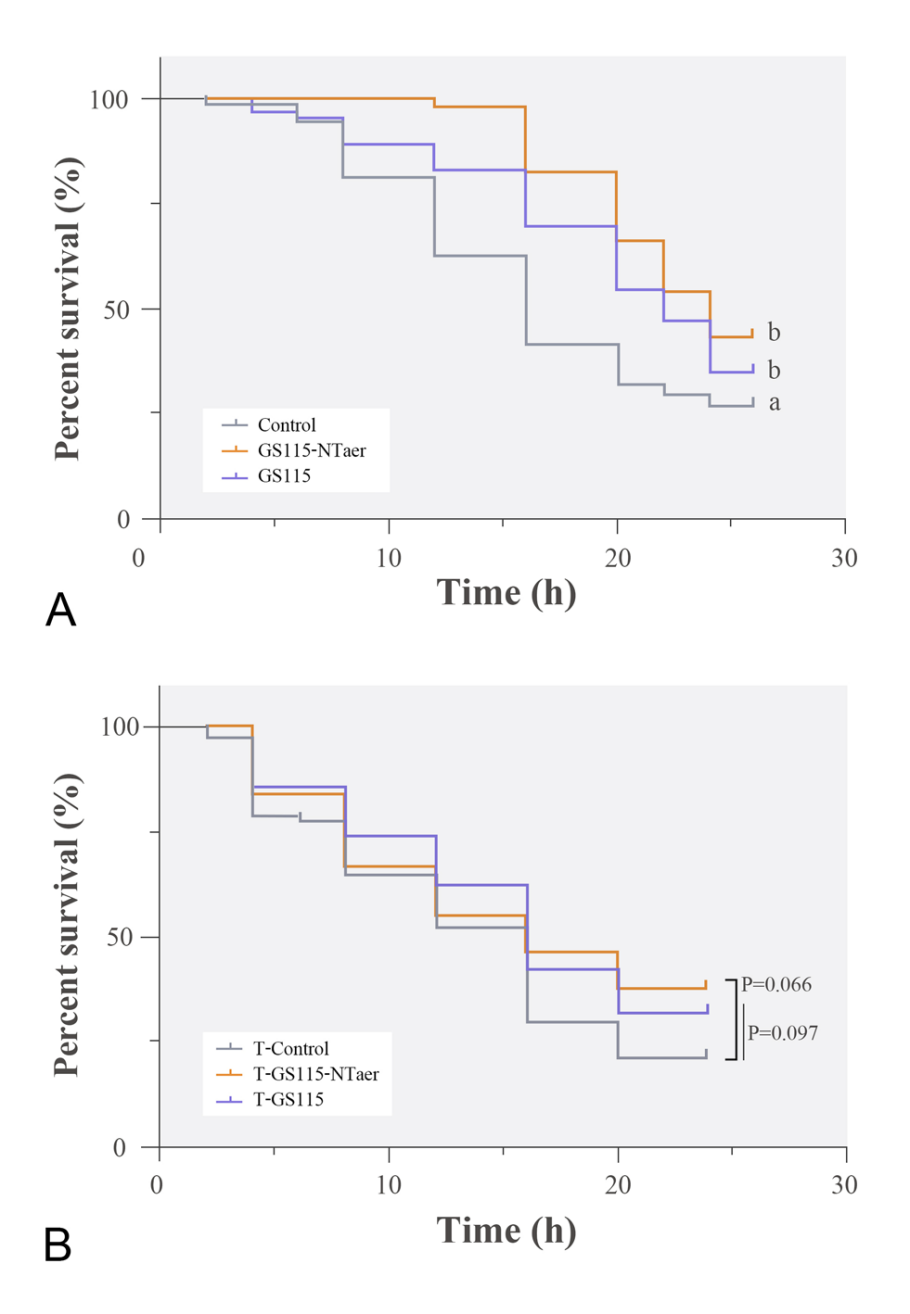
Survival rate of GF zebrafish. **A** Survival curve of GF zebrafish which directly immersed with GS115 or GS115-NTaer strain after challenged with *A. veronii*, the untreated group was used as control. **B** Survival curve of germ-free zebrafish which immersed with intestinal microbiota induced by control, GS115, or GS115-NTaer vaccine diet after challenged with *A. veronii*. Groups with different letters indicate significant differences (*P* < 0.05), *P* between 0.05 and 0.1 was considered trend

The susceptibility indexes after vaccine immunization were selected to evaluate the innate immune response and the contribution of the intestinal microbiota to the resistance of pathogens in a GF zebrafish model. GS115 and GS115-NTaer recombinant strains directly immersed with GF zebrafish resulted in a significant up-regulation in the relative expression of intestinal tight-connecting *TJP-1a*. Also, genes related to innate immunity were activated to varying degrees. Thus, the GS115 treatment group significantly down-regulated the relative expression of LYZ, while significantly up-regulating the relative expression of *C3a* gene when compared with the control group. The expression of these two genes did not change significantly when compared with group GS115-NTaer. Also, there was not any significant change in gene expression of *C4* in group GS115 compared with the other two groups. In the GS115-NTaer group, the relative expression of *C4* gene increased significantly compared with the controls, but there was not any significant change compared with group GS115. The relative expression of *LYZ* and *C3a* in the GS115-NTaer group did not have any significant changes when compared to the other groups (Fig. 7A). In terms of resistance to bacterial challenge, the untreated control group had the lowest survival rate with 26.67%; the survival rate of the GS115-NTaer treated group was significantly higher, and the RPS was 22.72%. Similarly, the survival rate of the GS115 treated group was also significantly higher than that of controls, with a RPS of 11.16% (Fig. 8A). When the intestinal microbiota induced by the control, GS115 or GS115-NTaer vaccined groups transferred to GF zebrafish, the expression of the tight connection gene and innate immune response showed an increase in the GS115-NTaer microbiota transferred group (T-GS115-NTaer). Furthermore, the up-regulation of *C4* was significantly different compared with the treatment by the intestinal microbiota induced by the control group (T-Control, Fig. 7B). After the larvae were challenged with *A. veronii*, the survival rates of GS115-NTaer and GS115 microbiota transferred groups had a increasing tendency compared with the T-Control group, and the RPS of T-GS115-NTaer and T-GS115 was 20.04% and 12.60%, respectively (Fig. 8B).

## Discussion

Vaccination is a commendable method to increase survival and to prevent aquatic animal diseases. During the development of aquatic animal vaccines, the choice of antigen has a great influence on the efficacy of the vaccine. Aerolysin was anticipated to be the most important antigen among the multiplicity of possible virulence factors produced by *Aeromonas* spp. Moreover, immunization with the toxin led to protection against the pathogen (Iacovache et al. 2015). A study demonstrated that *A. hydrophila* aerolysin could be used as a new target for drug development because of antivirulence strategies (Dong et al. 2021). Aerolysin was one of the best antigens, but the recombinant protein retains hemolytic activity. Aerolysin mutant NTaer in this study that lost its hemolytic activity was obtained by truncating expression, and the removed part (area) was mainly domain 1. This exerts an important role in receptor binding, and was involved in dimerization (Iacovache et al. 2015). The hemolysis of sheep red blood cells did not occur even at high concentrations of aerolysin mutant NTaer, which indicates the safety of this protein as an antigen. *P. pastoris* was selected as the antigen expression host and vaccine delivery system. Already in aquaculture, *P. pastoris* has been used as a host to express iridovirus major capsid protein for largemouth bass (Yao et al. 2022), recombinant tilapia growth hormone (Acosta et al. 2007) and antimicrobial peptide Nk-lysin of tilapia (Chen et al. 2022a, b). However, all of the above-mentioned proteins were expressed using AOX1 promoters that required methanol induction. This limited the application of the yeast epression system in animals. In this study, constitutive expression promoter P_GAP_ and secretory signal peptide sp23 were used in combination. This combined strategy achieved success in the high-level constitutive expression of leech hyaluronidase after screening various combinations of promoters and signal peptides (Huang et al. 2020). In addition, yeast components, such as cell-wall polysaccharides, have been well known to possess immunostimulatory activity (Zhou et al. 2023). So yeast may have adjuvant properties. Therefore *P. pastoris* constitutive secretory expression aerolysin mutant NTaer may well be used as an oral vaccine.

Evidence from evolutionary studies suggested that immunocompetence is resource-limited and, therefore, expected to be weighed against other resource-demanding processes, such as growth (Galarza et al. 2021). However, in this study, there was no significant difference in growth performance between immunized and control groups. This is consistent with previous studies of manipulation of immunity in growth performance of other animals, possibly because the amount of energy required for the immune response is much lower than that of tissue growth (Cheynel et al. 2019). Therefore, this study demonstrated that the *P. pastoris* composition expressed NTaer can stimulate an effective immune response to prevent disease without affecting the growth performance of fish.

The intestinal mucosa, which is continually exposed to antigens from food, microbiota or oral vaccine, was the major barrier to defense against potentially harmful factors. The intestinal mucosal barrier is composed of physical, chemical, immune and microbial barriers (An et al. 2022). The intestinal tight junction protein expression was significantly promoted after oral vaccination of the fish. The integrity of this intestinal physical barrier helps maintain the homeostasis of the internal environment. Lysozymes are a part of the intestinal chemical barrier and can destroy cell walls to lyse microorganisms. The content of lysozyme in the vaccinated group was significantly increased in this study; this could be beneficial for defense against pathogenic microorganisms (Ferraboschi et al. 2021). The intestinal immune barrier included the innate and adaptive immune systems; vaccines worked by stimulating an effective immune response. Complements played a crucial role in guarding against pathogen invasion. It was found that C3 has an important effect on grass carp infected with *A. hydrophila*, and protects tissues from bacterial damage (Meng et al. 2019). In this study, the significant increase of C3 and C4 in the vaccinated group indicates the activation of innate immunity. Genes related to antigen ingestion, processing, presentation and the cellular immune response were changed significantly not only in the local gut but also in the systemic immune tissue, such as the spleen. This may have happened because of the local innate cells releasing cytokines into the circulation to enable coordinated local mucosal and systemic immune responses immediately following vaccine administration (Roth et al. 2022). Given the spatial and temporal differences, the relative expression of genes in the gut and spleen was a little different. In the spleen, the significantly higher expression of macrophage-expressed gene (*MPEG*) 1 indicated an increase in the activity of macrophages, and perhaps also increased activity of a subpopulation of B cells (Ferrero et al. 2020). The higher expression of *CD4*, *CD8,* and T cell receptor (*TCR-α*) indicated that the vaccine activated the T cell immune response effectively. Moreover, significantly higher expression of transcription factors *T-bet* and *GATA3* indicated the activation of Th1 and Th2 immune responses in the fish. In the adaptive immune response—in addition to the cellular immune response—the humoral immune response was also effectively activated. This phenotype was reflected in the markedly increased levels of systemic antibody IgM and mucosal antibody IgZ.

Through two-way communication, both evolution and adaptive, mutually beneficial relationships were established to form a microecosystem between host intestinal mucosa and intestinal microbiome, that is the intestinal microbial barrier (Takiishi et al. 2017). Since the intestinal microbiome may be affected by a variety of factors (Luan et al. 2023), several existing studies were unable to define homeostasis or dysregulation by the presence or absence of specific microbial species (Lee et al. 2022). In this study, the host intestinal microbiota was changed significantly after vaccination; GS115-NTaer significantly reduced the abundance of potential opportunistic pathogens (Proteobacteria) in the gut. In agreement with this study, tilapia vaccination was determined to affect the structure of the gut mucosal microbiota and their metabolites (Wu et al. 2022). Therefore, the indices of core gut microbiota with differences in the function and ecological niche of fish gut ((Firmicutes + Fusobacteriota + Bacteroidota)/Proteobacteria) was evaluated for use as the biomarker to determine the steady state of the intestinal microbial barrier. This could reflect the health state of the host (Li et al. 2024). In human studies, a phylogenetically diverse core gut microbiota may also indicate functionally indispensable ecosystem service providers of the host in health or disease conditions (Metwaly et al. 2022; Zhang et al. 2015). After being immunized with GS115-NTaer, the ratio of (Firmicutes + Fusobacteriota + Bacteroidota)/Proteobacteria was significantly increased, indicating that the intestinal microbiota of the vaccinated group was more stable and the host was healthier.

The good protective effect after vaccine immunization is the result of a variety of factors. It is well known that stimulating a specific immune response is one of the essential elements of a successful vaccine. In this study, a GF zebrafish model was used to demonstrate that innate immunity and vaccine-optimized intestinal microbiota were also key factors for successful immunity, although it is difficult to identify the precise contribution of each factor.

## Conclusion

In conclusion, the aerolysin mutant NTaer was a safe and effective antigen. The recombinant *P. pastoris* GS115-NTaer strain was used to construct and evaluate the potentiality as an oral vaccine. This study verified that this oral vaccine had no significant adverse effects on the growth performance of zebrafish. Moreover, the vaccine played a significant role in the activation of effective immune protection, which was reflected by the up-regulation of the expression of immune-related genes, significantly increased IgM and IgZ antibody levels, optimization of intestinal microbiota structure, and most importantly, improved the survival rate of fish after challenge with *A. veronii* Hm091. Further research and application of this potential oral vaccine will contribute to the green prevention and control of *A. veronii* in aquaculture.

## Data Availability

All data generated during this study are presented in this published article, and are also available from the corresponding authors upon reasonable request.
